# The Reconstruction of Causal Networks in Physiology

**DOI:** 10.3389/fnetp.2022.893743

**Published:** 2022-05-03

**Authors:** Moritz Günther, Jan W. Kantelhardt, Ronny P. Bartsch

**Affiliations:** ^1^ Max Planck Institute for Meteorology, Hamburg, Germany; ^2^ Institute of Physics, Martin-Luther-University Halle-Wittenberg, Halle, Germany; ^3^ Department of Physics, Bar-Ilan University, Ramat Gan, Israel

**Keywords:** time series analysis, network physiology, Granger causality, bivariate phase rectified signal averaging, sleep apnea, heartbeat, respiration, brain-wave amplitudes

## Abstract

We systematically compare strengths and weaknesses of two methods that can be used to quantify causal links between time series: Granger-causality and Bivariate Phase Rectified Signal Averaging (BPRSA). While a statistical test method for Granger-causality has already been established, we show that BPRSA causality can also be probed with existing statistical tests. Our results indicate that more data or stronger interactions are required for the BPRSA method than for the Granger-causality method to detect an existing link. Furthermore, the Granger-causality method can distinguish direct causal links from indirect links as well as links that arise from a common source, while BPRSA cannot. However, in contrast to Granger-causality, BPRSA is suited for the analysis of non-stationary data. We demonstrate the practicability of the Granger-causality method by applying it to polysomnography data from sleep laboratories. An algorithm is presented, which addresses the stationarity condition of Granger-causality by splitting non-stationary data into shorter segments until they pass a stationarity test. We reconstruct causal networks of heart rate, breathing rate, and EEG amplitude from young healthy subjects, elderly healthy subjects, and subjects with obstructive sleep apnea, a condition that leads to disruption of normal respiration during sleep. These networks exhibit differences not only between different sleep stages, but also between young and elderly healthy subjects on the one hand and subjects with sleep apnea on the other hand. Among these differences are 1) weaker interactions in all groups between heart rate, breathing rate and EEG amplitude during deep sleep, compared to light and REM sleep, 2) a stronger causal link from heart rate to breathing rate but disturbances in respiratory sinus arrhythmia (breathing to heart rate coupling) in subjects with sleep apnea, 3) a stronger causal link from EEG amplitude to breathing rate during REM sleep in subjects with sleep apnea. The Granger-causality method, although initially developed for econometric purposes, can provide a quantitative, testable measure for causality in physiological networks.

## 1 Introduction

Causality is an ambiguous term and there are numerous philosophical, sociological, statistical, physical and information-theoretic approaches to define causality [Granger (1980); Hlaváčková-Schindler et al. (2007); Pearl and Mackenzie (2018)]. Although classified as statistical rather than a causal concept by some authors [see e.g., Hamilton (1994); Pearl (2009) for well-founded arguments], Granger causality [“*G*-causality”, Granger (1969)] provides a generally accepted operational framework to investigate causal interactions in time series. Going back to an idea by Norbert Wiener [Wiener (1956); therefore also “Wiener-Granger Causality”], Clive Granger was first to apply a linear regression model to probe whether a process *X* has a causal relationship with another process *Y* {or whether *X* can forecast *Y* [Hamilton (1994)]}. Limitations of this linear approach and prominent non-linear extensions of *G*-causality are discussed in detail by Hlaváčková-Schindler et al. (2007).

In the field of physiological time series analysis and in particular when probing for physiological interactions, *G*-causality plays a major role along with entropy-based measures [Schulz et al. (2019)], phase synchronization analysis and symbolic dynamics [Müller et al. (2016)]. Indeed, *G*-causality is frequently used in the emerging field of Network Physiology [Bashan et al. (2012)] to investigate the network interactions between multiple physiological systems involved in cardiovascular/cardiorespiratory control [Porta and Faes (2015); Schulz et al. (2013)] and heart-brain coupling [Faes et al. (2015)]. It is becoming a standard tool in neuroscience to identify directed functional interactions in the brain [Hesse et al. (2003); Bressler and Seth (2011); Seth et al. (2015)]. However, *G*-causality was initially developed for economic time series [Granger (2004)], which are usually shorter (regarding their number of samples) and sampled at lower frequencies than physiological time series. This must be considered when using *G*-causality for physiological applications. Notably, the condition of “instantaneous causality” [Granger (1969)] may, in contrast to economic data, not be present in physiological data because typical sampling rates are higher than the delay time of the causal relationships [Lin et al. (2016)]. Furthermore, there are forms of coupling between physiological systems that coexist but operate at different time scales [Bartsch et al. (2014); Bartsch and Ivanov (2014)]. Therefore, in order to identify physiological interactions for fast as well as very slow processes, the original model must be extended and *G*-causality computed at different temporal resolutions.

An often discussed drawback of *G*-causality for practical implementations is the necessity of data being stationary, which is usually not the case for physiological recordings. Workarounds range from simply differentiating the data or analyzing shorter (“quasi-stationary”) time windows to more complex methods utilizing an adaptive recursive least-square algorithm [Hesse et al. (2003)] or applying spectral density matrix factorization of the Fourier and wavelet transforms [Dhamala et al. (2008)] — with each method having its own pros and cons [Bressler and Seth (2011)].

An alternative, simple yet powerful method, which does not require stationarity to investigate interactions and causal relations between time series, is Bivariate Phase Rectified Signal Averaging (BPRSA) analysis [Schumann et al. (2008)]. While originally developed as a mono-variate method to study quasi-periodic oscillations in non-stationary signals [Bauer et al. (2006b)] and quantify cardiovascular risk [Bauer et al. (2006a)], its bivariate extension has been applied to assess spontaneous baroreflex sensitivity [Müller et al. (2012)], and, more recently, to analyze maternal-fetal heart rate coupling [Montero-Nava et al. (2020)].

Because BPRSA is, in contrast to *G*-causality, a model-free approach to study inter-relationships and causality in physiological signals, in this paper we aimed for a systematic comparison of both methods. This will be done in Part A after a proper introduction of each method. In particular, we will elaborate on their strengths and weaknesses, and present statistical tests to probe for significant interactions. The analysis is done with a focus on possible applications in physiology. A corresponding example regarding physiological networks during sleep will be presented in Part B of the paper.

## 2 Part A: Methods for Causality Analysis

### 2.1 Differentiating Between Direct and Indirect Links

**FIGURE 1:**
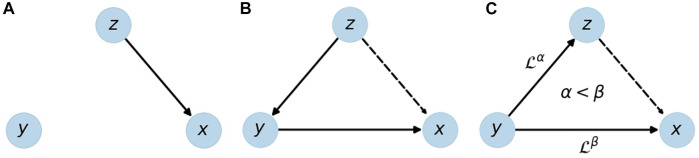
Illustration of a direct link **(A)** and indirect links **(B,C)** from source *z* to target *x*. An indirect link can be due to a causal chain *z* → *y* → *x* **(B)** or due to time delayed effects of *y* on *x* and *z* with a longer time delay towards *x*. Solid edges represent direct links, dashed edges the resulting indirect links; L is the time lag operator.

In physiological networks an important problem is to distinguish direct from indirect links. Figure 1 depicts direct and indirect links in simple three-node networks. In all three cases, the node corresponding to the source signal *z* seems to influence the target node, i.e., signal *x*. While there is a direct link from *z* to *x* in subfigure (a), there is only an indirect link in subfigure (b), mediated by signal *y*. Subfigure (c) shows another form of an indirect link, where the link between *z* and *x* is purely due to the common influence of *y* on both signals. Note that a time lag, indicated by the operator L , is important only in case (c). If the time lag from *y* to *z* was longer than the lag from *y* to *x* (i.e., *α* > *β*), the indirect link would change its direction and point from *x* to *z* instead.

There are more complex and mixed cases e.g., direct and indirect links between the same two nodes, and coexisting links [Bartsch and Ivanov (2014)], but the ones shown in Figure 1 are the most basic setups, and in the following they will be used to test and compare *G*-causality and BPRSA. By studying results for modeled data, we show that *G*-causality is more appropriate to distinguish these setups within a certain range of detection limits.

### 2.2 Method 1: Granger Causality

A simplified definition of Granger causality is: “Variable *z* Granger-causes (*G*-causes) variable *x* if knowledge about *z* improves the forecast of *x*” [Granger (1969)]. This reflects our common understanding of cause and consequence: The cause must precede the consequence in time and if *z* has no effect on *x*, we do not call it causal. This idea is formalized in the framework of autoregressive (AR) processes.

Under fairly general conditions a random process can be described by an AR model of order *p* [see, e.g., Lütkepohl (2005)]. Consider two AR models of the time series *x*_*t*_, one including and one excluding information on *z*_*t*_,

$$x_{t}=\overset{p}{\underset{i=1}{\sum }}\left(\phi_{i}^{\left(1\right)}x_{t-i}\right)+w_{t}^{\left(1\right)};\;\;\text{STD}\left(w^{\left(1\right)}\right)=\sigma^{\left(1\right)},$$ (1)

$$x_{t}=\overset{p}{\underset{i=1}{\sum }}\left(\phi_{i}^{\left(2\right)}x_{t-i}+\psi_{i}^{\left(2\right)}z_{t-i}\right)+w_{t}^{\left(2\right)};\;\;\text{STD}\left(w^{\left(2\right)}\right)=\sigma^{\left(2\right)}.$$ (2)

Equation 1 models the value of *x* at time *t* as a weighted average over its own past plus a white noise process *w* with zero mean and standard deviation (STD) *σ* ^(1)^. The weighting factors *ϕ*_*i*_ can be obtained by minimizing the error term *w* ^(1)^. The past of *x* carries information about its own future, but this information is necessarily incomplete due to the statistical nature of *x*. The better *x* can be described (forecast) from its own past, the lower the standard deviation *σ* ^(1)^ of the residual *w* ^(1)^.

Equation 2 additionally considers information about *z*. If this information helps to model (forecast) *x*, then the standard deviation *σ* ^(2)^ will be reduced compared to *σ* ^(1)^. The *G*-value *G*_*z*→*x*_ is a measure for the improvement of the forecast of *x* by including *z*, and therefore a measure of causality,

$$G_{z\to x}=\text{ln}\frac{\sigma^{\left(1\right)}}{\sigma^{\left(2\right)}}.$$ (3)

*G*_*z*→*x*_ quantifies *G*-causality, however it remains unclear whether 1) an obtained *G*-value is significantly different from zero, and 2) the link is direct or indirect.

To resolve issue 1), one can test the null hypothesis that all coefficients $\psi_{i}^{\left(2\right)}$ of Eq. 2 are practically zero, i. e., *G*-causality does not exist. This is done by assuming an F or chi-squared distribution, given that the estimators of the least square method coefficients are asymptotically normally distributed [Neusser (2011)], and estimating the probability of a non-zero mean value. Because no other variables (such as *y*) are taken into account, this is a *pairwise analysis* method, which can identify links from *z* to *x* in all three cases of Figure 1.

In order to differentiate between direct and indirect links, i.e., to resolve issue 2), a *conditional analysis* is necessary. Consider the two extended AR models,

$$x_{t}=\overset{p}{\underset{i=1}{\sum }}\left(\phi_{i}^{\left(3\right)}x_{t-i}+\tau_{i}^{\left(3\right)}y_{t-i}\right)+w_{t}^{\left(3\right)}\;\;\text{ STD}\left(w^{\left(3\right)}\right)=\sigma^{\left(3\right)},$$ (4)

$$x_{t}=\overset{p}{\underset{i=1}{\sum }}\left(\phi_{i}^{\left(4\right)}x_{t-i}+\tau_{i}^{\left(4\right)}y_{t-i}+\psi_{i}^{\left(4\right)}z_{t-i}\right)+w_{t}^{\left(4\right)}\;\text{STD}\left(w^{\left(4\right)}\right)=\sigma^{\left(4\right)}.$$ (5)

Equations 4, 5 are the same as Eqs. 1, 2, except that the past of *y* is added to both. Hence, *σ* ^(4)^ is lower than *σ* ^(3)^ if and only if *z* adds information that is not already provided by *y*. Therefore, the conditional analysis will not show *y*-conditional *G*-causality

$$G_{z\to x}^{\left(y\right)}=\text{ln}\frac{\sigma^{\left(3\right)}}{\sigma^{\left(4\right)}}$$ (6)

for the indirect *z* → *x* links shown in Figures 1B,C, but only for the direct link in Figure 1A. Besides, cases (b) and (c) can be distinguished by a pairwise analysis of *z* and *y* (disregarding *x*). This idea can be extended to a set of arbitrarily many variables, but for the scope of this work three variables (time series *x*_*t*_, *y*_*t*_, and *z*_*t*_) are sufficient.

Stationarity is an important prerequisite for the AR framework, because the process’ characteristics (i.e., the coefficients *ϕ*_*i*_, *ψ*_*i*_, *τ*_*i*_) must not change over time. A stationary process is a process with a constant mean and a finite covariance function that is invariant to shifts in time. This requirement is problematic, because many physiological signals are inherently non-stationary [Ivanov et al. (1996); Goldberger et al. (2002)]. Here, we probe stationarity with the Augmented Dickey-Fuller (ADF) test, which is widely accepted [Paparoditis and Politis (2018)] and based on AR modeling, so that it operates in the same framework as *G*-causality analysis.

In summary: *G*-causality is based on the improvement of a forecast by including additional data from other signals, and it can only be applied to stationary data. It can be used to distinguish between the three setups shown in Figure 1, if pairwise analysis and conditional analysis are applied. With *G*-causality the existence of a causal link can be decided as yes/no question with a statistical test, and quantified with the *G*-value.

### 2.3 Method 2: Bivariate Phase Rectified Signal Averaging

While the *G*-causality approach is sensitive to non-stationarities, the Phase Rectified Signal Averaging method [PRSA, Bauer et al. (2006b)] and its bivariate version [BPRSA, Schumann et al. (2008)] have been developed to study noisy, non-stationary signals. The methods are especially suitable for quasi-periodic time-series, where perturbations reset the signal phase at random times. The BPRSA approach can easily be extended to an arbitrary number of signals. The idea is to align windows of the target signal *x* that are in the same phase with respect to one or more trigger signals (*y* and *z*) and average over all these windows. The procedure is described in detail in Schumann et al. (2008), see also Bauer et al. (2006b,a, 2009); here we only provide a brief overview.

The easiest and standard way to define trigger events from signal *z* is to consider all positions, where the signal increases, i. e., $z_{t_{\nu }}>z_{t_{\nu }-1}$ for a trigger event at time *t*_*ν*_. We denote all *m* trigger points by *t*_*ν*_, *ν* = 1, …, *m*. Any other criterion that returns a Boolean value (trigger event or no trigger event) is possible, including criteria that are based on multiple signals. Each trigger event at *t*_*ν*_ leads to an anchor point $x_{t_{\nu }}$ of the target signal. Then, windows of width 2*L* are chosen around each anchor point $x_{t_{\nu }}$ ,

$$\left\{x_{t_{\nu }-L},x_{t_{\nu }-L+1}\ldots ,x_{t_{\nu }+L-1}\right\}.$$ (7)

The resulting BPRSA function for a potential *z* → *x* link is the point-wise average of *x*_*t*_ in all of these *m* windows,

$${\text{BPRSA}}_{j}=\frac{1}{m}\overset{m}{\underset{\nu =1}{\sum }}x_{t_{\nu }+j},\;j=-L,-L+1,\ldots ,L-1.$$ (8)

The choice of anchor points is supposed to guarantee that in each window the index *j* = 0 is at a similar phase of the physiological process. The average over all windows is an in-phase superposition and therefore insensitive to non-stationarities (that are slower than the time scale *L*) and artifacts.

If there is no relation between *z* and *x*, and *m* is large, the resulting BPRSA time series will be constant everywhere, however with statistical fluctuations. This also happens if the choice of trigger points is not appropriate to reveal a relation between the signals, because it does not reflect the underlying processes. Any significant deviation from a constant value for any BPRSA_*j*_ must be interpreted as a relation between *z* and *x*, but not necessarily in the sense of *G*-causality. A positive (negative) peak in the BPRSA time series indicates the positive (negative) influence of a trigger event in *z* on *x*, or—in case the peak is at a negative index *j*—from *x* on *z*. Thus, BPRSA yields temporal information on cause and effect, just like *G*-causality. However, we would like to note that—unlike G-causality analysis—BPRSA does not model the time series data in any way, but relies on an averaging procedure assuming that the selected trigger event criterion is suitable for the relation between the considered signals and that the Central Limit Theorem holds. Therefore, it is expected that longer data are needed for a reliable identification of causality relations with BPRSA.

Since no test for the statistical significance of such a relation has yet been proposed^1^, we have studied and compared four tests. The null hypothesis is that there is no causality between *z* and *x*. If this holds, the trigger points are randomly distributed and each of the BPRSA_*j*_ values, Eq. 8, is an average over independent random numbers and thus normally distributed due to the central limit theorem.

The following statistical tests are considered and compared:

1) The one-sided Kolmogorov-Smirnov test [Massey (1951)] measures the difference between the probability density functions of the BPRSA and a normal distribution, providing a *p*-value for the null hypothesis.
2) The two-sided Kolmogorov-Smirnov test [Massey (1951)] compares the distribution of the real BPRSA values with the distribution of BPRSA values for random trigger points (i.e., disregarding the trigger signal *z*), also providing a *p*-value for the null hypothesis.
3) The Anderson-Darling test [Anderson and Darling (1952)] works similar to this idea, but introduces a weight function that increases the importance of the tails of the distribution. This is particularly useful if deviations from normality appear as abnormally high or low values instead of deviations in the middle of the bell-shaped curve.
4) The Shapiro-Wilk test [Shapiro and Wilk (1965)] is the most powerful of these tests according to Razali and Wah (2011). It is based on variance analysis and compares the variance of a normal distribution with the estimated variance of the sample.

In summary: The existence of causality in the sense of BPRSA can be tested by checking whether the BPRSA_*j*_ values are normally distributed. In addition, the peak height can provide quantitative information on the link strength. Compared to *G*-causality, this method is less sensitive to non-stationarities, and it is a model-free approach. However, BPRSA cannot distinguish direct from indirect links unless more evolved trigger criteria could be established for a conditional analysis.

### 2.4 Results and Discussion: Comparison of *G*-Causality and BPRSA Causality

#### 2.4.1 Pairwise Analysis

With the tools presented above, both *G*-causality and BPRSA offer ways to test pairwise causality of two signals. In the first step, we quantified the detection limits. To this end, two signals of 1/*f* ^*α*^ noise with *α* = 0.5 were created by the Fourier filtering method [Makse et al. (1996); Bashan et al. (2008)]. Starting with white noise, the power spectrum was rescaled to follow 1/*f* ^0.5^ behavior, and—back in the time domain—the values were rescaled to have unit variance. These original noise signals are called *o*_1_ and *o*_2_. The signals *z* and *x* are defined as

$$z=o_{1},\;x=L^{3}z\times q+o_{2}\times \left(1-q\right),\;0<q<1.$$ (9)

Here, L shifts the series by one time unit, so that $L^{3}$ shifts it by three units; the number three was arbitrarily chosen. The unitless number *q* quantifies how much *x* is influenced by *z*. This setup was designed to test what influence is necessary for causality to be detected by the different methods. The length of the time series was varied from 2^6^ = 64 to 2^16^ = 65,536 samples. The length of the BPRSA time series was chosen to be 2*L* = 30, triggering on a rising signal [Bauer et al. (2006a,b); Schumann et al. (2008)]. Especially for the short time series large parts of the data had to be discarded due to overlap of window and boundary. While choosing a shorter window length might result in less discarded data, the averaged BPRSA time series would become shorter and therefore impair the quality of the statistical tests. The boundary effects are negligible for longer time series. For all tests the null hypothesis was no causality. In our setup *z* causes *x* with varying strength *q*. Therefore, a test yields the correct result if the null hypothesis is rejected. We rejected the null hypothesis for *p*-values lower than 0.05.

**FIGURE 2:**
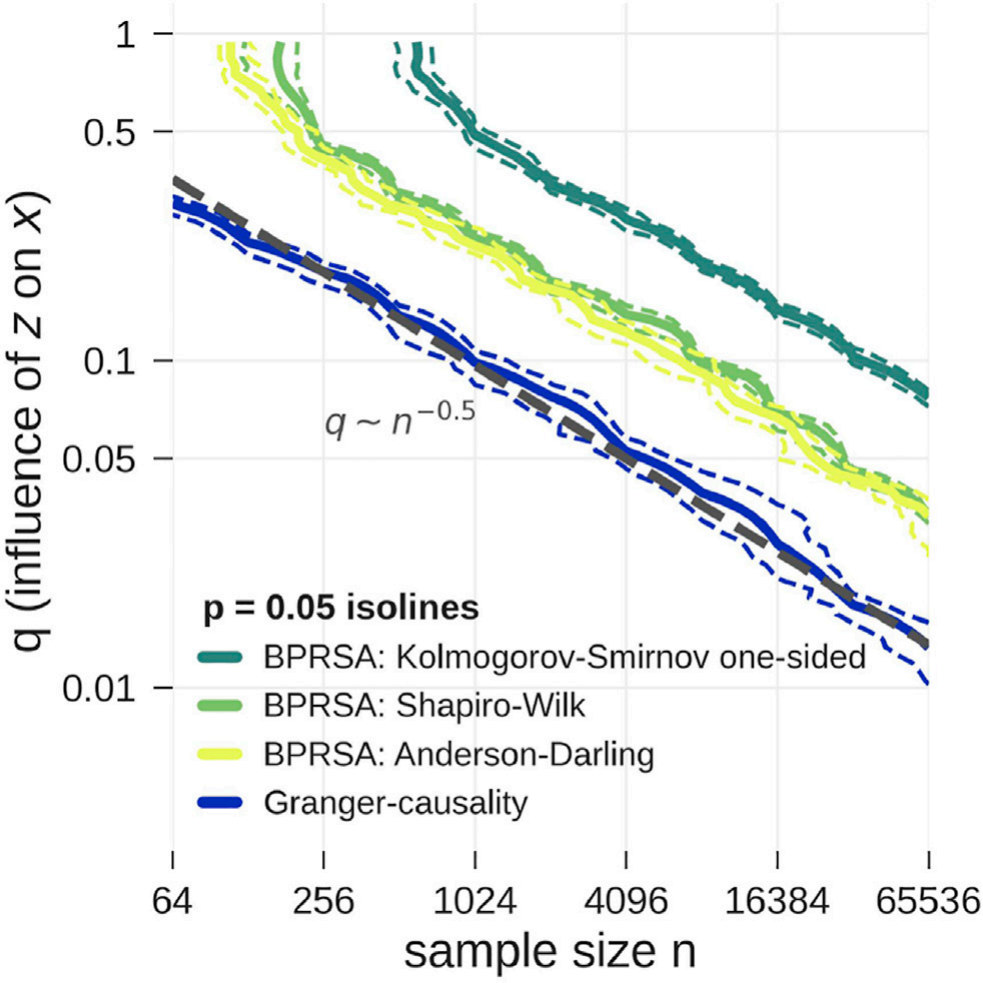
Model data according to Eq. 9 is tested for pairwise causality with statistical tests applied to *G*-values (blue) and BPRSA values (yellow and green). The plot shows the *p* = 0.05 isolines for each test as function of the influence strength *q* and the length of the time series (*x*_*t*_ and *z*_*t*_). The isolines’ 5 and 95% confidence interval are marked by dashed lines, computed from a bootstrap procedure. The tests correctly reject the null hypothesis *above* the shown isolines towards the top right corner of the plot. Clearly, the lowest detection limit is achieved by *G*-causality (applying the F-test). BPRSA causality with the Anderson-Darling test (3) and the Shapiro-Wilk test (4) is less sensitive by a factor of two to three in the influence strength *q* as compared to *G*-causality. BPRSA causality with the one-sided Kolmogorov-Smirnov test (1) is even less sensitive by another factor of 2, while the two-sided Kolmogorov-Smirnov test (2) only yielded the correct result in the top right corner of the plot and is therefore not shown. There seems to be a power-law relationship between the critical *q*-value as function of the sample size, *q* ∼ *N* ^−0.5^.

Figure 2 shows the dependency of the threshold for the identification of existing causality on sample size and link strength for all proposed tests. All experiments were averaged over 20 realizations in order to get a statistically reliable result. The confidence intervals are given by the 5th and 95th percentile of a bootstrap distribution, which is obtained from 100 random samples. For each sample, we drew 20 out of the 20 realizations, allowing individual realizations to be picked multiple times.

In the limit of large sample sizes and strong influence *q*, all tests correctly identified causality (upper right corner of Figure 2). In the limit of small sample size and weak influence *q*, all tests failed (lower left corner of Figure 2). The detection limit is given by the *p* = 0.05 isoline. For small sample sizes (64 points or less) BPRSA is unable to detect causality, no matter how strong the link, possibly because a non-negligible amount of data near the boundaries is discarded. The two-sided Kolmogorov-Smirnov test failed to detect causality in all cases and is therefore not shown in Figure 2. The one-sided version turned out more powerful, but remained weaker than the Anderson-Darling test and the Shapiro-Wilk test. These results are in line with the findings of Razali and Wah (2011). The *G*-causality test detects causality for smaller sample sizes and weaker influences than all BPRSA tests.

In the calculations for Figure 2, BPRSA causality methods turned out to need about two times more computational effort than *G*-causality, and all BPRSA tests failed for short time series. As mentioned above, it is expected that BPRSA needs longer data for a significant result, since it relies on the Central Limit Theorem. For nonstationary data, BPRSA can still be applied, but even longer data would be needed, since the non-stationarities must cancel out in its averaging procedure. Furthermore, BPRSA is dependent on the choice of the trigger criterion. Only if a trigger criterion that fits to the physiological process is chosen, relationships can be established. Without prior knowledge, several trials with all kinds of criteria need to be done in order to probe for BPRSA causality. *G*-causality does not require such procedure, but also cannot be used to test for different kinds of time-varying relations between the considered signals in a system with non-stationary dynamics. Therefore, BPRSA can still be advantageous if specific hypotheses on the nature of the signals’ relations exist or if the signals’ relations change in time.

Both methods provide information on the direction of coupling, while methods such as cross correlation analysis or cross-spectral analysis are symmetric in the sense that a coupling of signal *x* with signal *z* is always also a coupling of *z* with *x*. BPRSA and *G*-causality both overcome this problem. We note that in Figure 2 a power-law relationship between the critical *q*-value as function of the sample size can be seen, however, a detailed study of this scaling is beyond the scope of the present work.

In summary, *G*-causality is a more powerful method than BPRSA causality. Still, the BPRSA method is more likely to be able to handle nonstationarities because of its natural strength in phase-aligning the signal parts. However, methods have been developed to overcome the stationary constraint and apply time-dependent AR-models [Ding et al. (2000)], which allow the definition of time-dependent *G*-causality [Hesse et al. (2003)]. We concluded that *G*-causality is better suited to detect causality in multivariate, stationary setups and continued our analysis with this method.

#### 2.4.2 Conditional Analysis

Consider the setup shown in Figure 1C. There is a relation between *z* and *x*, but it only exists because of the common influence of *y*. While BPRSA cannot distinguish between a direct influence of *z* on *x* or a common driver *y* on *z* and *x*, with *G*-causality these cases can be separated. This is a real strength of *G*-causality, and in this subsection we are testing the limits of such detection. One way to formalize the setup is by extending Eq. 9 to three variables, with *o*_1_, *o*_2_ and *o*_3_ again being independent 1/*f* ^0.5^ noise signals,

$$\begin{array}{cc} y & =o_{2};\\ z & =L^{2}y\times q_{y\to z}+o_{1}\times \left(1-q_{y\to z}\right);\;0<q_{y\to z}<1\\ x & =L^{4}y\times q_{y\to x}+o_{3}\times \left(1-q_{y\to x}\right);\;0<q_{y\to x}<1. \end{array}$$ (10)

**FIGURE 3:**
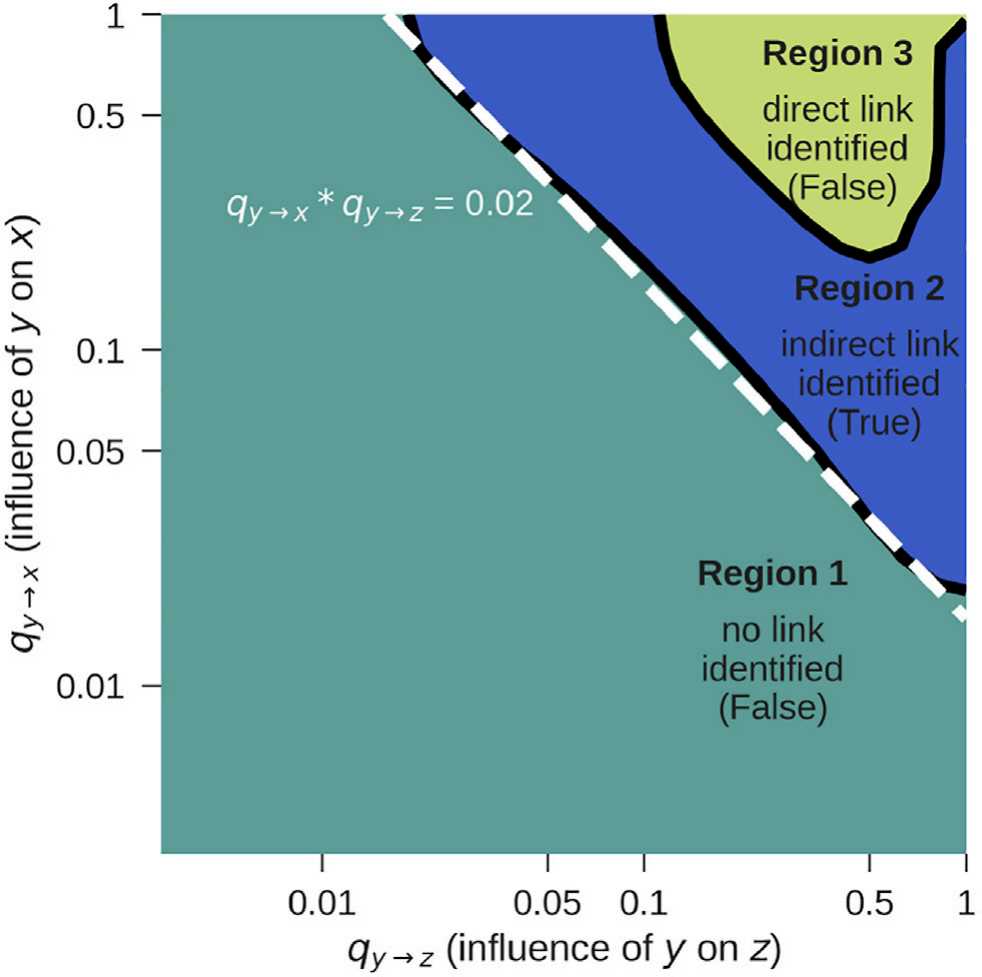
*G*-causality F-test for *z* → *x* for the setup from Figure 1C, according to Eq. 10. The correct result is obtained in Region 2. For further details see text.

We performed pairwise and conditional analysis, see Eqs. 1–5, and tested for *G*-causality with an F-test for signals of sample size 2^15^ = 32,768 and varying influences *q*_*y*→*z*_, *q*_*y*→*x*_. The results, averaged over 20 realizations, are shown in Figure 3.

We identified three regions:

• **Region 1**: Both, the pairwise and the conditional test do not reject the null hypothesis that there is no *G*-causality. This would lead to the conclusion that there is neither a direct nor an indirect link, which is false. The reason for the false negative result is that the influence coefficients *q*_*y*→*z*_ and *q*_*y*→*x*_ are too weak. Longer time series will shift this detection limit towards the lower left corner of the figure.
• **Region 2**: While the pairwise test rejects the null hypothesis, the conditional analysis does not. This leads to the conclusion that there is a *z* → *x* link, which can only be indirect. This is indeed true and corresponds to the case shown in Figure 1C.
• **Region 3**: Both the pairwise as well as the conditional test reject the null hypothesis, indicating that there is a direct *z* → *x* link, which is false. If *q*_*y*→*z*_ and *q*_*y*→*x*_ become so strong that *x* and *z* are both very tightly coupled to *y*, *G*-causality is mistakenly detected. In practice, this limitation rarely applies, because signals are usually not free of noise and not coupled so strongly.

If *y* is known, the method works for log  *q*_*y*→*z*_ + log  *q*_*y*→*x*_ = log (*q*_*y*→*z*_ ⋅ *q*_*y*→*x*_) ≥ log (0.02) = −1.7 (dashed line, Region 2), as long as the link is not extremely strong (Region 3). If the influence is too weak, no causality will be detected at all. If it is extremely strong, the indirect link will be mistaken for a direct link.

In practice, measurements will always be limited to certain variables, and there is no way to exclude the possibility of external variables *y* that constitute a common cause to the measured signals. For this reason, there can practically never be certainty if a detected link is direct or due to a common source. One approach to overcome this is to include as many variables in the model as possible, which is, however, problematic because an increasing number of parameters must be estimated in this procedure. Prior knowledge on the modeled process can improve the interpretation of the results.

## 3 Part B: Reconstruction of Causal Physiological Networks

In this part, conditional *G*-causality is applied to detect direct physiological couplings between heart rate, breathing rate, and EEG amplitude during sleep. We will show in the following that the couplings among these physiological systems differ between groups of young healthy subjects, elderly healthy subjects and patients with obstructive sleep apnea (OSA). OSA is the temporary, complete or partial disruption of normal respiration during sleep, caused by a reduced tonus of upper airways muscles [Dempsey et al. (2010)]. The increased negative intrathoraic pressure upon inspiration causes the upper airways to collapse, which results in a drop of blood oxygen and increase in blood carbon dioxide levels. This leads to an arousal from sleep, followed by recovery of normal respiration [Penzel et al. (2003b)].

### 3.1 Methods

Physiological time series were derived from polysomnography (PSG) measurements that were recorded in several European sleep laboratories between September 1997 and April 2000 as part of the EU-project SIESTA [Klosch et al. (2001)]. Before any analysis, we chose 36 young, healthy subjects with excellent signal quality (young control group—YC, aged 29 ± 6), 36 elderly, healthy subjects (elderly control group—EC, aged 51 ± 10) and 43 age-matched, elderly subjects with an apnea-hypopnea index (AHI) of at least 10 per hour (OSA group, aged 51 ± 9). AHI is the mean number of apnea and hypopnea events per hour when considering a full-night sleep. Genders are distributed approximately equally in the YC (17 male, 19 female) and EC group (18 male, 18 female), but the OSA group consists mostly of male participants (38 male, five female). We address this in the results section. For each subject, we derived:

• **Instantaneous heart rate H** as the inverse RR-interval, i.e., the time between two successive heart beats.
• **Instantaneous breathing rate B** from the inverse interval between two extrema of the raw respiration signal. The raw respiration signal was chosen for each subject individually as the best-quality signal out of effective oronasal airflow and stretch belts placed around abdomen and thorax.
• **EEG** α instantaneous amplitude by applying a bandpass filter on the EEG signal using the α frequency band 7.8–15.6 Hz.

**FIGURE 4:**
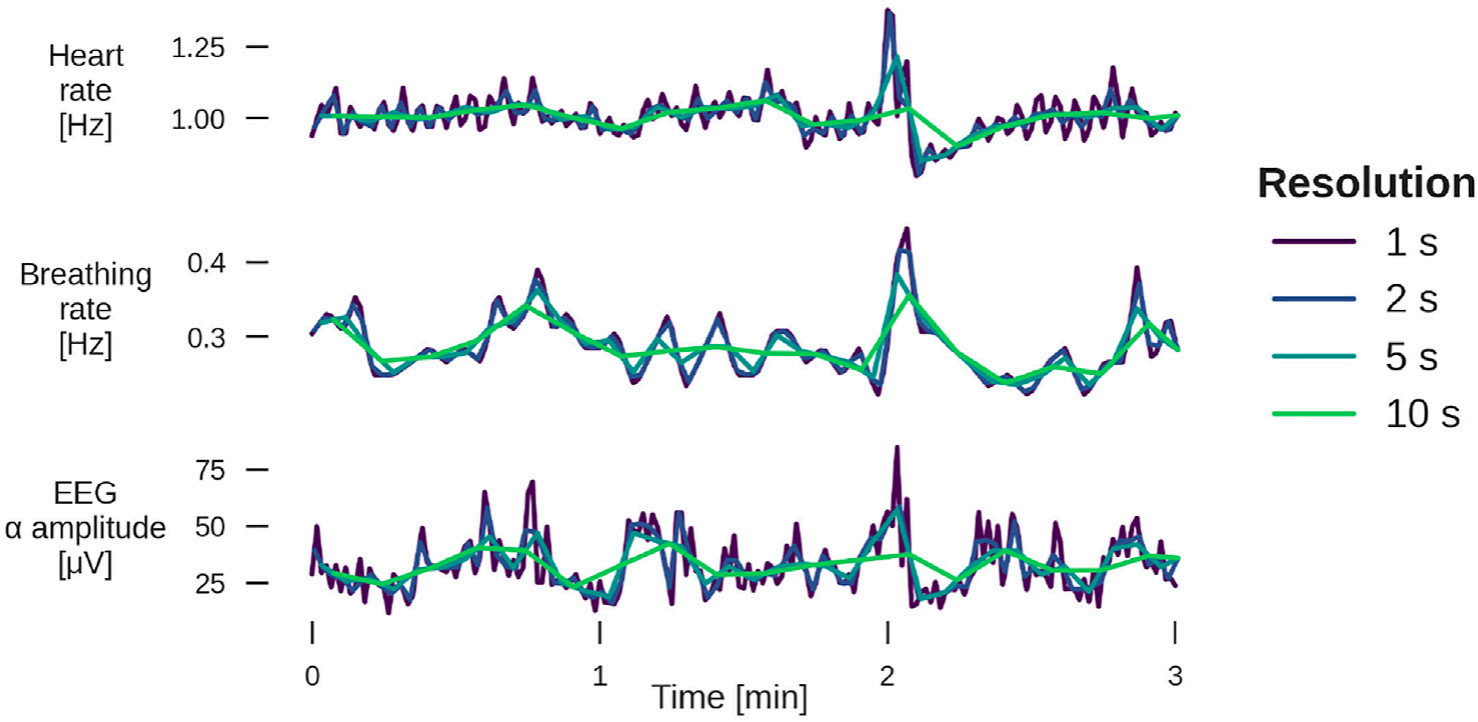
Example of 3 min segments from a YC subject for heart rate (top panel), breathing rate (middle), and EEG α amplitude (bottom) for four different temporal resolutions. The sleep stage is light sleep. For examples from other sleep stages, see Supplementary Figures S1, S2.

Subsequently, signals H and B were interpolated to 1 Hz resolution, for EEG α, averages over non-overlapping windows of one second length were taken. The resulting three time series were all sampled at 1 Hz, and were averaged to resolutions of 2, 5, 10, 15, 30, and 60 s (i.e., 0.5, 0.2, 0.1, 0.067, 0.033, and 0.017 Hz) for further analysis (see Figure 4).

For each block of 30 seconds, sleep stages were scored by a sleep technician on the basis of the PSG signal following the rules by Rechtschaffen and Kales [Hobson (1969)]: light sleep (stages 1 and 2—LS), deep sleep (stages 3 and 4—DS) and REM sleep. Each triple of time series (H, B and EEG α) was partitioned into patches of continuous sleep of the same sleep stage, typically several minutes, and then normalized to zero mean and unity variance.

Since stationarity is a crucial precondition for *G*-causality analysis, a stationarity test must precede further analysis. We based our algorithm on the widely accepted Augmented Dickey-Fuller Test [ADF test, Paparoditis and Politis (2018)]. On the one hand, only stationary patches can be used for *G*-causality analysis, on the other hand the percentage of usable patches should be maximized in order to obtain the best possible statistics. A trade-off can be achieved through variation of the model order. For each continuous patch of the same sleep stage, all three signals of the node triple (i.e., heart rate, respiration rate and EEG amplitude) are tested for stationarity on a model of order 5. If all of them are stationary, the *G*-value is calculated according to Eq. 6. If at least one is nonstationary, the same procedure is repeated with model order 4, then with model order 3. If the process can still not be modeled as stationary at model order 3, the patch is split in half and the same procedure is applied to both shorter patches, motivated by the fact that shorter time series are more likely to be sufficiently free of trends and variability in variance and autocorrelations. A stopping condition is set when the patches reach a length of less than six times the time series resolution (i.e., 90 s length is the lower limit for time series of 15 s resolution), because this is the minimum length required for an AR model of order five. If this condition is met, the data is considered nonstationary and discarded.

**FIGURE 5:**
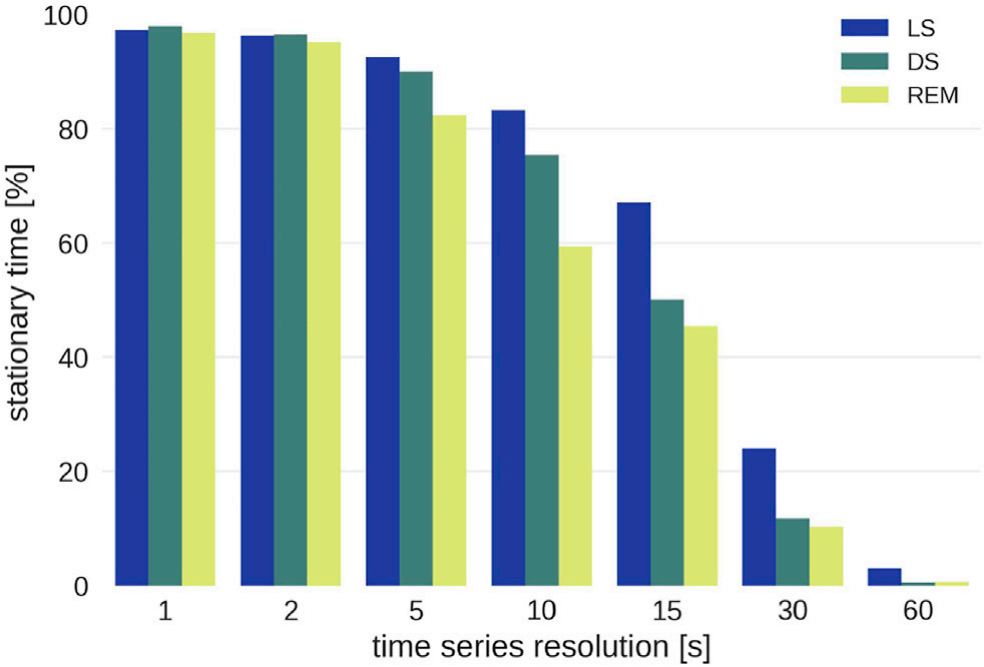
Percentage of stationary sleep episodes as function of the time scale for the three different sleep stages. Note that wake epochs were excluded from the analysis because of insufficient statistics.

Of all the data that are potentially available, the percentage of stationary patches is shown in Figure 5. It does not make sense to analyze data for resolutions broader than 15 s as most of these patches do not contain stationary data. This is mainly because of two reasons: Firstly, there are too few sufficiently long sleep stage epochs for resolutions > 15 s, and secondly, windows with an equal amount of data points but lower temporal resolutions cover longer sleep episodes, which are less likely to be stationary (i.e., compare high resolution 1 s time series with low resolution 60 s data). In our datasets, the second reason is dominant.

Even though resolutions above 15 s are of interest for research on long-term correlations, they cannot be included because there is not enough stationary data. For each group the conditional *G*-values (Eq. 6) for each time series resolution and each sleep stage were averaged and weighted by the length of the patch that they were calculated from in order to account for the varying length of sleep stages.^2^

**FIGURE 6:**
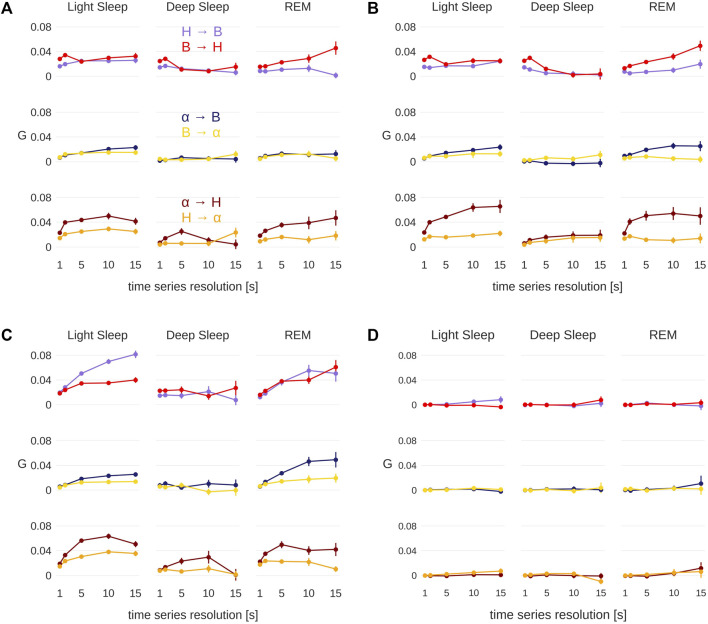
Pairwise conditional *G*-causality for the three groups [YC—**(A)**, EC—**(B)**, OSA—**(C)**] and surrogate data **(D)**. Error bars represent the standard error and were calculated using a bootstrap method [Efron and Tibshirani (1993), see text for more details].

Error bars were calculated using a bootstrap method [Efron and Tibshirani (1993)]: Out of all *G*-values that comprise a data point, a new set of *G*-values was randomly drawn. This set is as large as the original one, but the same *G*-value can be picked several times, while others are not included in a particular sample. Overall, 100 such samples were drawn and for each sample the (unweighted) mean was calculated. The standard deviation of these means is an estimate for the standard deviation of the actual mean, the standard error, and is plotted as error bar in Figure 6.

Each analyzed data patch consists of only tens to hundreds of data points, which is not enough to reliably detect weak links with the *G*-causality test (see Figure 2). Therefore, we compare our results with surrogate data that allow for an alternative way to validate *G*-causality: If the average *G*-value obtained for a particular group and pair of signals is different from the *G*-value of the surrogate data, *G*-causality can be assumed. For our surrogate analysis, the respiration rate, heart rate and EEG amplitude data were taken from three different subjects, so that no *G*-causality can be expected between any of the three signals [Toledo et al. (2002); Bartsch et al. (2007)]. An alternative method would be the adjusted-amplitude Fourier transform (AAFT) method [Theiler et al. (1992); Lavanga et al. (2020)].

### 3.2 Results

**FIGURE 7:**
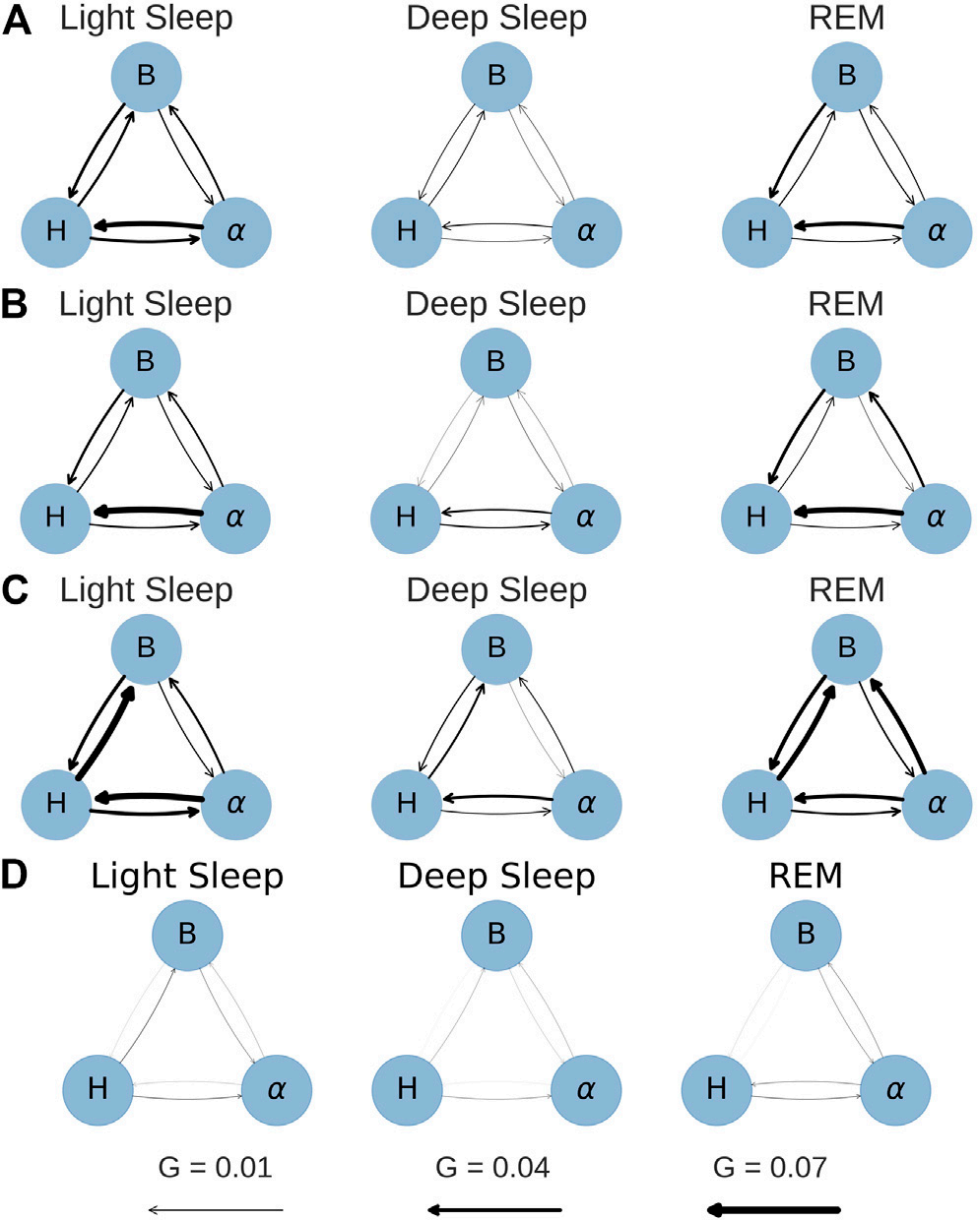
Network plots for conditional *G*-causality at a time series resolution of 10 s. The arrow width is directly proportional to the link strength (*G*-value). **(A)** YC. **(B)** EC. **(C)** OSA. **(D)** Surrogate.

The results for all groups and the surrogate data are shown in Figure 6. The figure shows two of the six combinations in each panel, grouped into pairs of signals for each sleep stage. The error bars represent the standard error calculated from the bootstrap procedure described above. Particularly for LS and REM, the difference in directionality increases for larger time windows (i.e., lower resolutions at 10 and 15 s), however, for 15 s the error bars are quite large in many cases. For this reason, time series with 10 s resolution were chosen to generate the physiological networks presented in Figure 7. Thus, the strength of the connection line between two nodes (“network link”) is proportional to the *G*-value at a resolution of 10 s. While Figure 7 presents less information than Figure 6, it is more helpful to find patterns and recognize important differences between the groups.

It is important to note that a time series resolution of 10 s does not mean that the relevant processes happen within 10 s. The model that is used to calculate the *G*-values comprises three to five past terms, which means that the processes occur in time windows of 30–50 s, but by default neglect causalities from processes with dynamics on time scales shorter than 10 s.

Subfigures (a), (b) and (c) in Figures 6, 7 show the results for the YC, EC and OSA group, respectively. The results for YC and EC are qualitatively very similar: The interaction between heart and respiration rate is symmetrical in light and deep sleep across all time scales and takes low to medium values (*G* < 0.04 at all times). This changes in REM sleep, where the causality from respiration to heart rate clearly dominates over the opposite direction, especially towards larger time scales. The *G*-value for the causality from respiration to heart rate at time scale 2 seconds is slightly increased (see Figures 6A,B only), which is an indicator of respiratory sinus arrhythmia (RSA), a well-known effect of modulation of the heart rate within the breathing cycle. During inspiration, the heart rate accelerates and it slows down during expiration [Angelone and Coulter (1964)]. This RSA peak disappears for REM sleep, which is in agreement with Bond et al. (1973), who describe “a total dissociation between respiration and rhythmic heart rate variability” during REM sleep. However, while RSA (which acts on relatively short time scales on the order of a breathing cycle) disappears in REM sleep, the causal relation from respiration to heart rate during REM sleep is shifted to longer time scales. The RSA peak is also absent in OSA subjects during non-REM sleep (see Figure 6C), possibly indicating reduced RSA for OSA subjects.

The coupling between EEG α amplitude and respiration rate remains, for all groups, constantly weak and symmetrical throughout all sleep stages and time series resolutions. The only deviation from this behavior is a slight increase in the causality from EEG α to respiration rate at larger time scales for EC and OSA subjects in REM sleep, which could be related to aging. Regarding the coupling between heart rate and EEG α, there is a clear dominance from EEG α to heart rate during light and REM sleep. This coupling almost completely vanishes during deep sleep, causing EEG α to heart rate coupling to become more symmetric.

The network plots 7(a) and (b) show the small differences between the YC and EC group. Furthermore, throughout all groups there is only little *G*-causality during deep sleep. This is in accordance with results of Bashan et al. (2012) and Bartsch et al. (2015) who show low network connectivity during deep sleep using a time delay stability approach to quantify interactions. At the same time, during deep sleep there is also a loss of long-term correlations in heart beats [Bunde et al. (2000); Penzel et al. (2003b)] as well as in respiratory inter-breath intervals [Schumann et al. (2010)]. Such long-term correlations, however, exist during light and REM sleep and are assumed to be due to influences from the sympathetic nervous system on cardiac and respiratory dynamics [Schmitt et al. (2009); Bashan et al. (2012)]. In contrast, deep sleep, which is considered the most restorative sleep stage [Dijk (2009)], is characterized by sympathetic withdrawal and greatly reduced influence of the autonomic nervous system on heart and respiratory dynamics. In our results, this is reflected by a more autonomous behavior of all three nodes for all groups. A careful comparison between the YC and EC group reveals that EEG α to heart rate coupling may slightly increase with age. For OSA patients this effect is even more pronounced and accompanied by an overall increase of coupling strength also between heart and breathing as well as EEG α to breathing, possibly indicating a decreased deep sleep quality because of sleep apnea.

In general, the OSA group follows similar sleep-stage patterns as the YC and EC groups. Distinct differences, however, can be seen in the overall strength of coupling: First and most strikingly, the *G*-causality from heart rate to respiration rate is much stronger during light and REM sleep than in the other two groups. This can be attributed to different relaxation speeds of heart and respiratory rate after apnea events. Following an episode of sleep apnea, heart and respiration rate are increased [Penzel et al. (2003a; 2016)], but the relaxation of the heart rate happens faster than the relaxation of the respiratory rate. This leads to a situation where changes in heart rate precede changes in respiratory rate and thus lead to a detection of increased *G*-causality, as indicated by higher *G*-values. Secondly, there is an increased *G*-causality from EEG α amplitude to breathing during REM sleep.

Figures 6D, 7D show results for the surrogate data. As expected, the *G*-value is very close to zero in all cases at all resolutions, with small deviations for the 15 s data point, which could be due to the large error bars at this resolution. These results can be used as a baseline showing that randomly assembled physiological data yield negligible *G*-values, and that any deviation from zero, as seen in all other sub-figures, are physiologically meaningful results.

Performing the same analysis without testing for stationarity yields slightly different results (see Supplementary Figures S4, S5). Values at short timescales are mostly unchanged, but it seems that the G-value is overestimated in some cases when ignoring the stationarity test. Additionally, the standard errors are larger, especially for longer time scales. Therefore, we conclude that only stationary data yield non-spurious network links when applying the *G*-causality method. The fact that the OSA group consists of mostly male subjects does not influence our results. We repeated the analyses of Figures 6, 7 excluding all female subjects from YC and EC, and obtained similar results (see Supplementary Figures S6, S7).

## 4 Conclusion and Outlook

In Part A, the causality methods BPRSA and *G*-causality were analyzed. Both methods are strong analysis tools to detect interrelations and causality in time series, but also have limitations. While BPRSA has rather weak testing methods and fails to distinguish direct from indirect links, the application of *G*-causality is limited due to the fact that it requires time series to be stationary (which is often not the case in physiology). For the setups investigated in this work, *G*-causality yielded better results than BPRSA causality tests. The *G*-value provides a measure of the strength of causality, and it can be computed for pairwise (bivariate) and conditional (multivariate) setups, where the latter includes additional information beyond the causing and the caused signal. Important for Network Physiology, this enables the distinction between direct and indirect links as well as links that arise from a common source signal. While (in contrast to BPRSA) the *G*-causality method is constrained by the stationarity condition, there are extensions to the method that circumvent the problem [Hesse et al. (2003); Dhamala et al. (2008); Bressler and Seth (2011)]. A particular, simple way to overcome this restriction is to split non-stationary data into shorter, stationary patches. Further investigations and alternative approaches to overcome the stationarity condition could be promising future research pathways.

In part B, the *G*-causality method was applied to the node triple consisting of heart rate, respiration rate, and EEG α amplitude, recorded from subjects with and without OSA. The *G*-value was calculated for time series resolutions between 1 and 15 s. Causal physiological networks were constructed based on these *G*-values. In all groups, strong coupling between respiration and heart rate and from EEG α to heart rate can be observed in light and REM sleep. In contrast, during deep sleep, the three nodes are practically “decoupled”, especially for the young group. This result supports earlier findings and the understanding of deep sleep as the sleep stage with lowest sympathetic tone. Because aging changes sympathovagal balance due to a reduced parasympathetic tone [Schmitt et al. (2009)], leading effectively to higher sympathetic activity, the *G*-causality coupling in deep sleep is slightly increased for the elderly and OSA groups. Apart from deep sleep, results are very similar for young and elderly healthy subjects, however, OSA subjects show some distinct differences. Compared to the other two groups, the most prominent difference is an increase in *G*-causality from heart rate to respiration rate in light and REM sleep due to different relaxation times of heart rate and respiration rate, which are both increased at the end of an apnea event. Disturbances in respiratory sinus arrhythmia during light and deep sleep and a stronger causal link from EEG α to breathing rate during REM sleep can also be observed in OSA subjects.

Our findings point to the conclusion that sleep of persons with sleep apnea is not only different with respect to breathing behavior, but also with respect to coupling mechanisms like respiratory sinus arrhythmia and deep sleep decoupling. Comparisons with surrogate data prove the significance of the obtained results. Overall, the application to causal networks in subjects with and without sleep apnea demonstrates the usefulness of *G*-causality as a measure for physiological coupling.

## Data Availability

The data analyzed in this study is subject to the following licenses/restrictions: All code is available at the following GitHub repository: https://github.com/moritz-g/causal_networks_physiology. Data can be obtained upon request from the SIESTA Group. Requests to access these datasets should be directed to www.thesiestagroup.com.

## Footnotes

1 Previous publications focus on the analysis of definitely existing relations [Bauer et al. (2006b,a); Schumann et al. (2008); Bauer et al. (2009, 2010)].

2 We note that theoretically *G*-values cannot be negative, because this would imply a reduction in predictive power caused by adding new information, which is impossible. In the worst case the new information could just be neglected, which should lead to a *G*-value of zero. However, because of numerical reasons the minimization algorithm used for the parameter calculation of the models can lead to negative *G*-values in few cases. Instead of artificially removing those cases they were kept because in other cases the algorithm slightly overestimates the *G*-value. Removing the negative values would introduce a bias towards higher *G*-values.
