# Supplementary material for: The Reconstruction of Causal Networks in Physiology
Source: Front Netw Physiol. 2022 May 3;2:893743. doi: 10.3389/fnetp.2022.893743 (PMC10013035; doi:10.3389/fnetp.2022.893743)
Supplement: Supplementary file 1 [file DataSheet1.PDF]

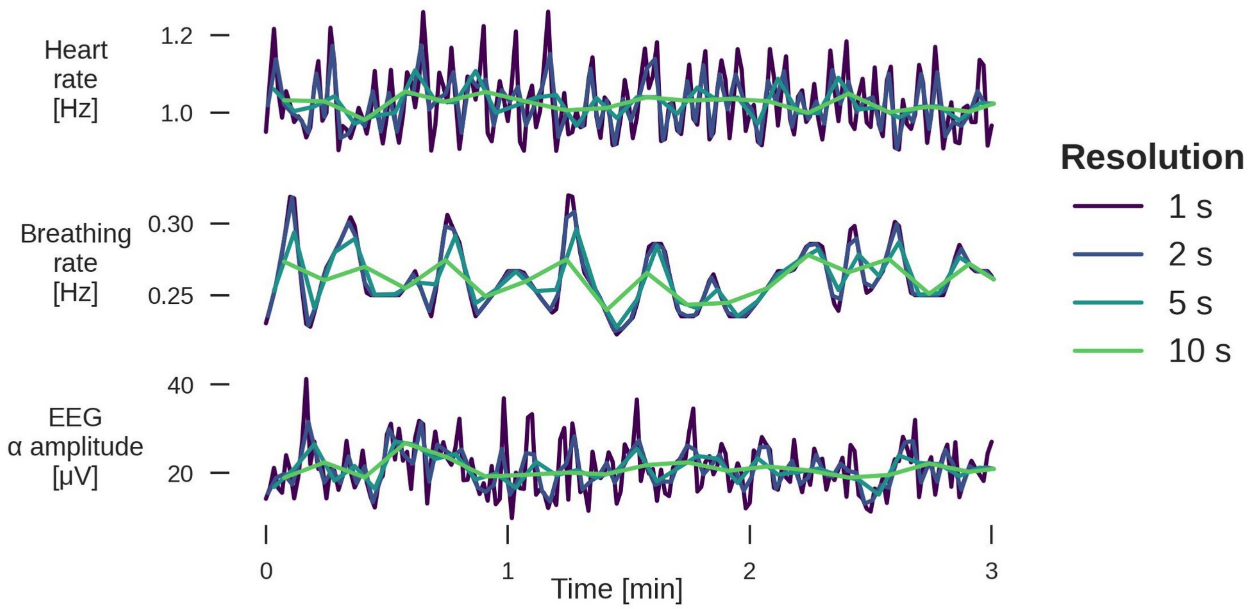

Fig. S1: Example of 3 min segments from a YC subject for heart rate (top panel), breathing rate (middle), and (c) EEG α amplitude (bottom) for four different temporal resolutions. The sleep stage is deep sleep.

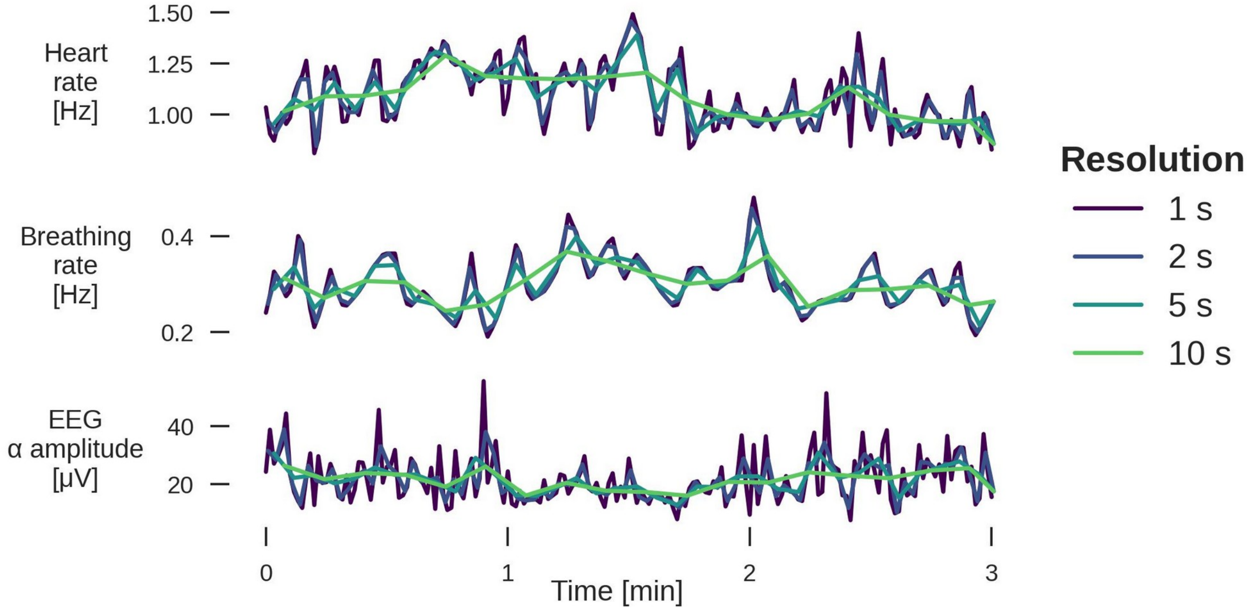

Fig. S2: Example of 3 min segments from a YC subject for heart rate (top panel), breathing rate (middle), and (c) EEG α amplitude (bottom) for four different temporal resolutions. The sleep stage is REM.

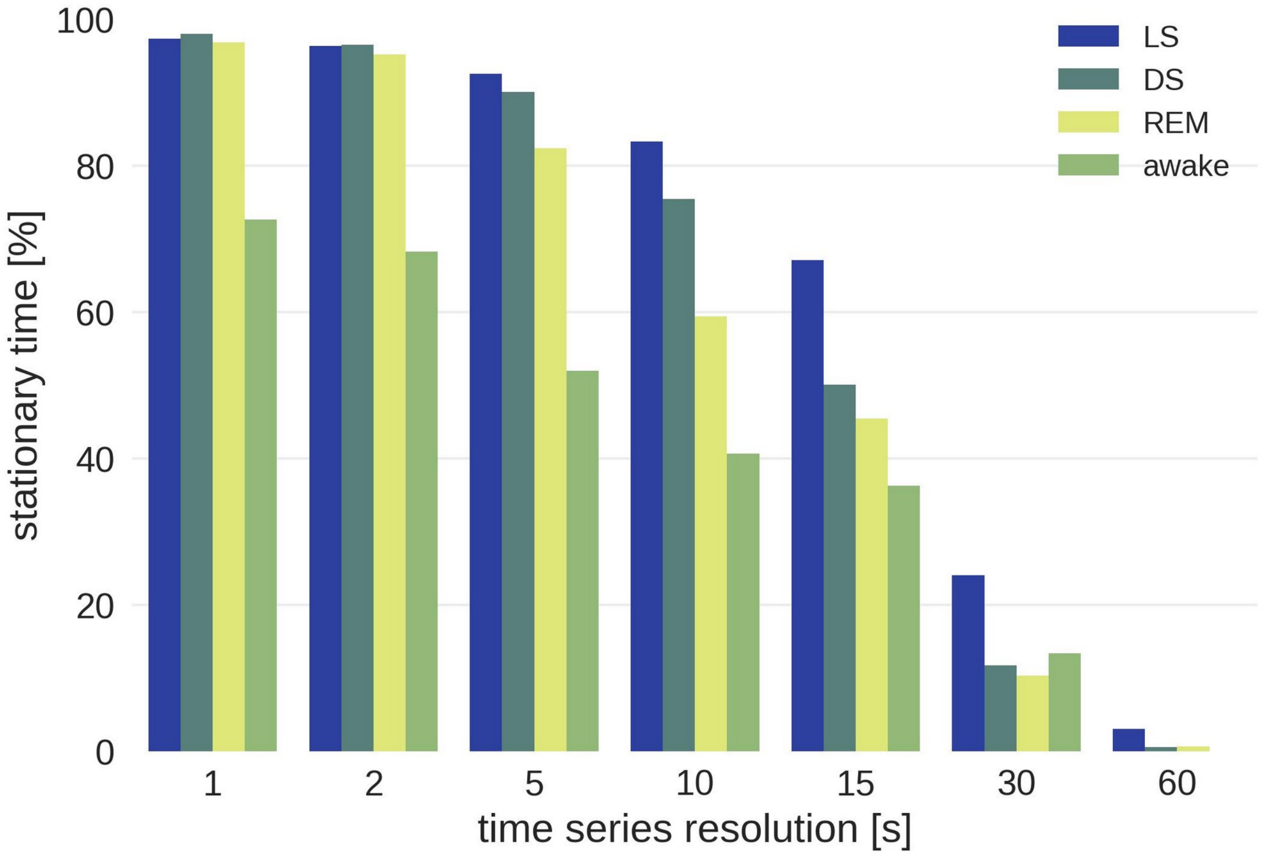

Fig. S3: Same as Fig. 5, but including the “awake” episodes.

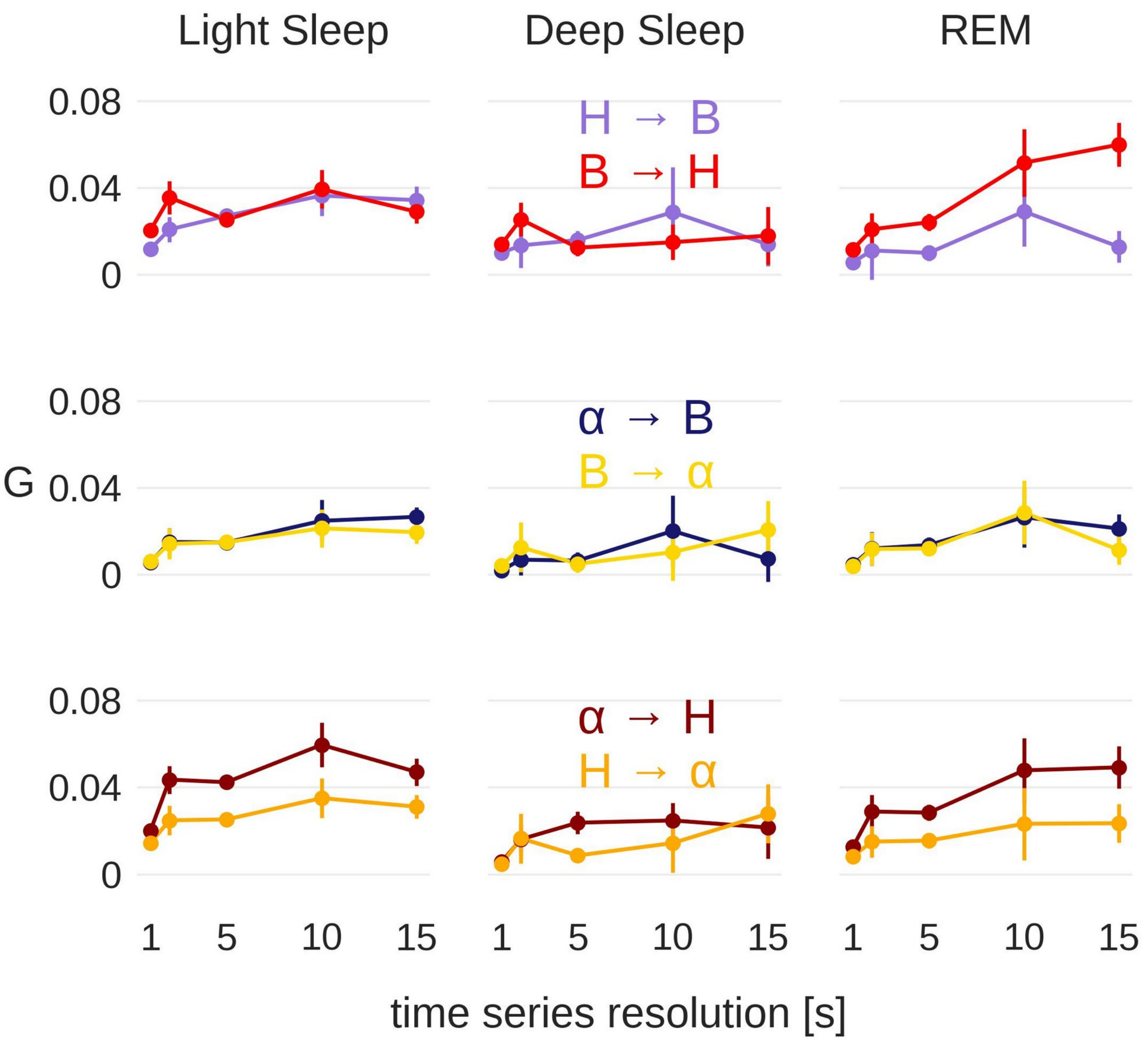

Fig. S4(a): Same as Fig. 6(a) (YC group), but including all data without discarding non-stationary segments.

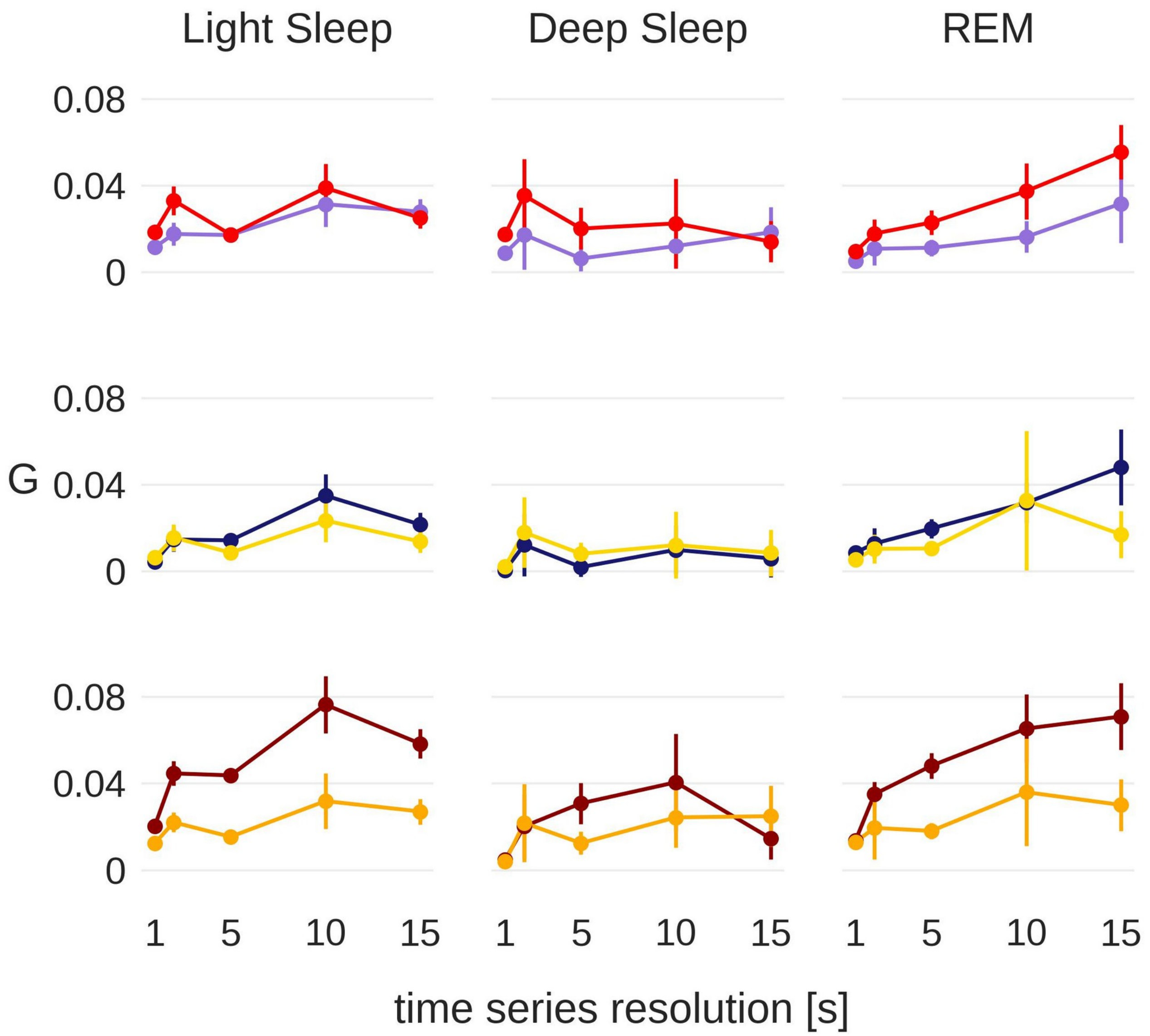

Fig. S4(b): Same as Fig. 6(b) (EC group), but including all data without discarding non-stationary segments.

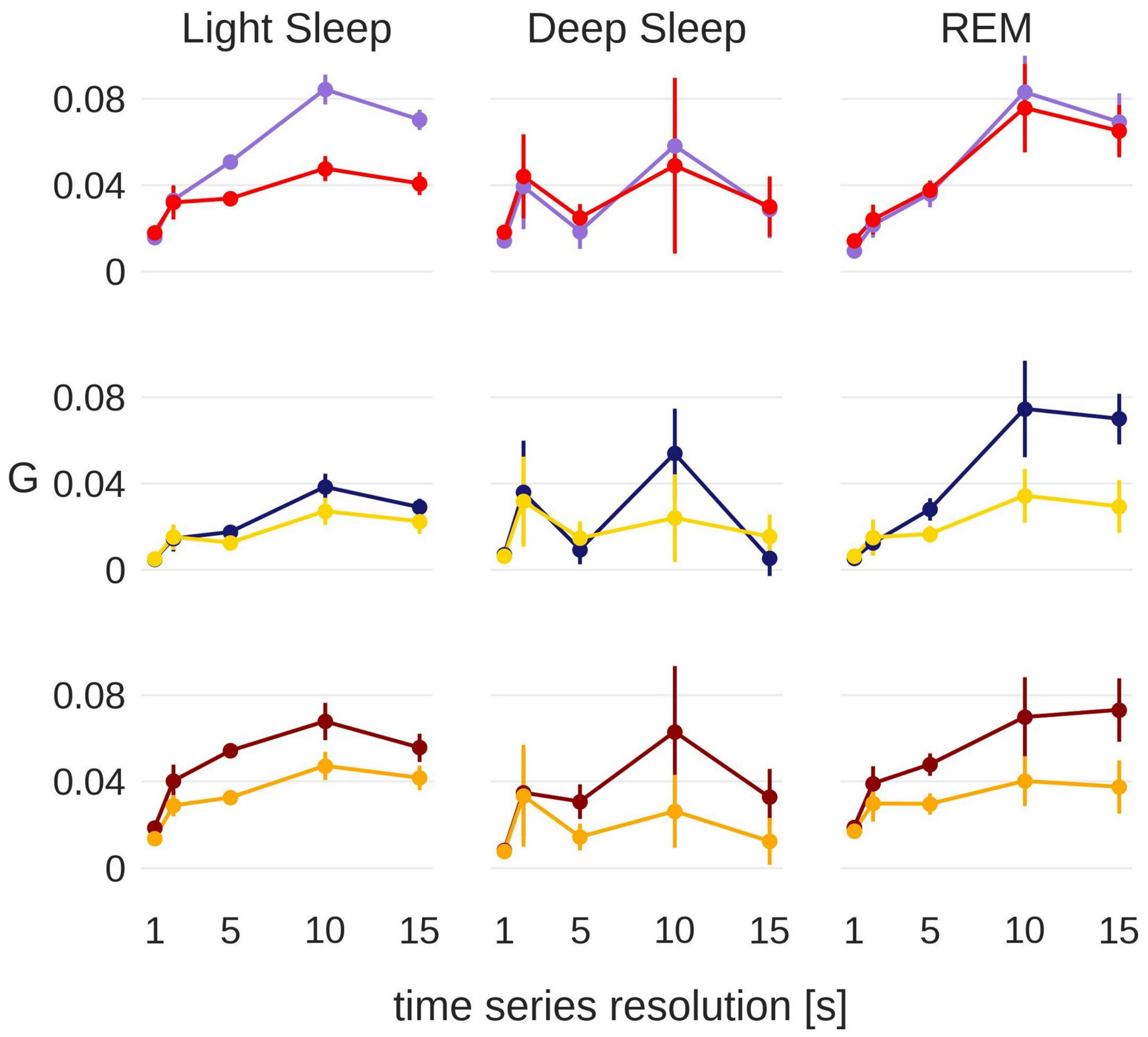

Fig. S4(c): Same as Fig. 6(c) (OSA group), but including all data without discarding non-stationary segments.

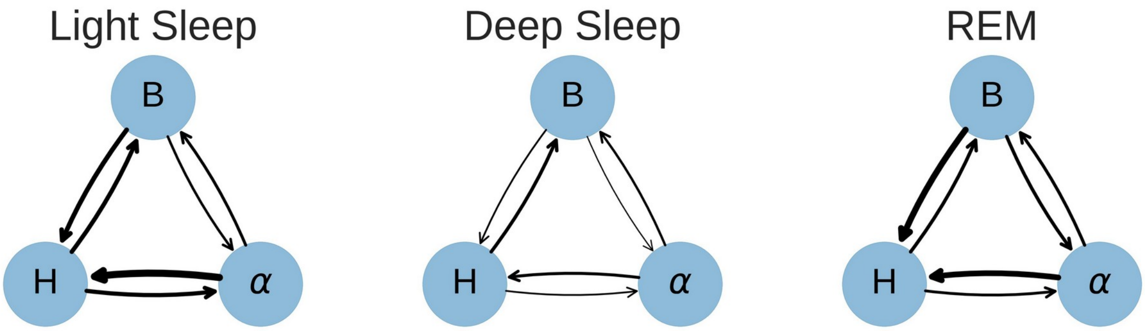

Fig. S5(a): Same as Fig. 7(a) (YC group), but including all data without discarding non-stationary segments.

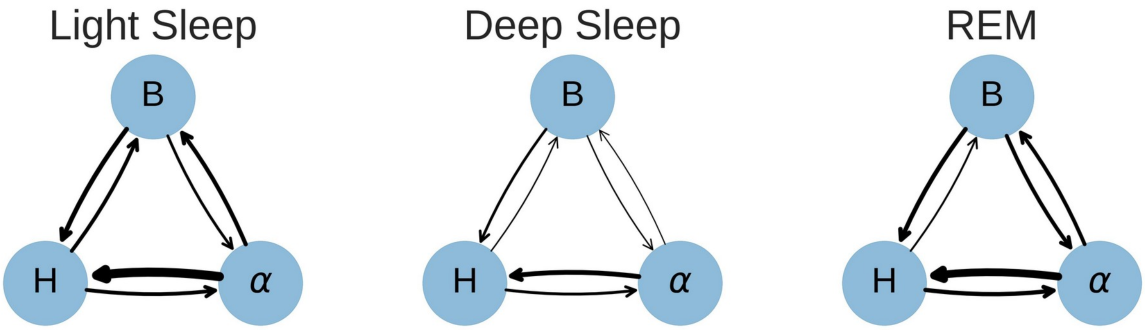

Fig. S5(b): Same as Fig. 7(b) (EC group), but including all data without discarding non-stationary segments.

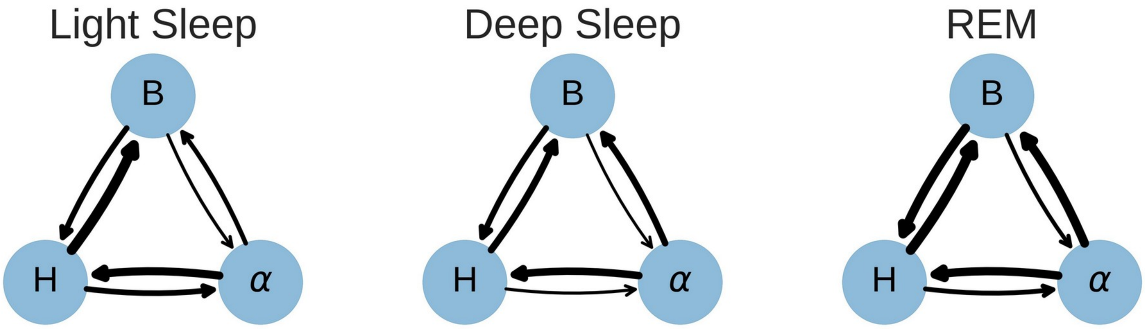

Fig. S5(c): Same as Fig. 7(c) (OSA group), but including all data without discarding non-stationary segments.

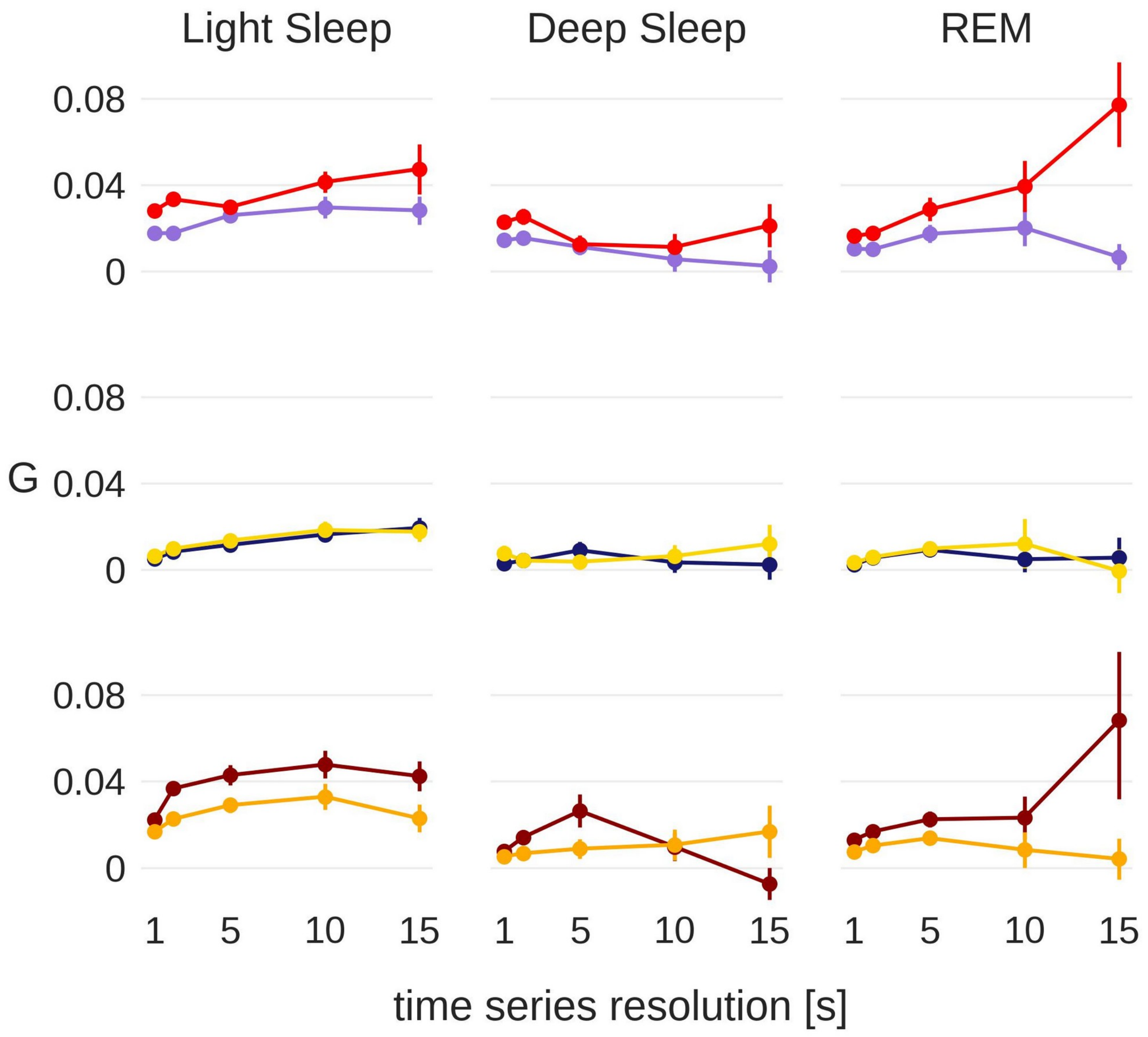

Fig. S6(a): Same as Fig. 6(a) (YC group), but only including male subjects.

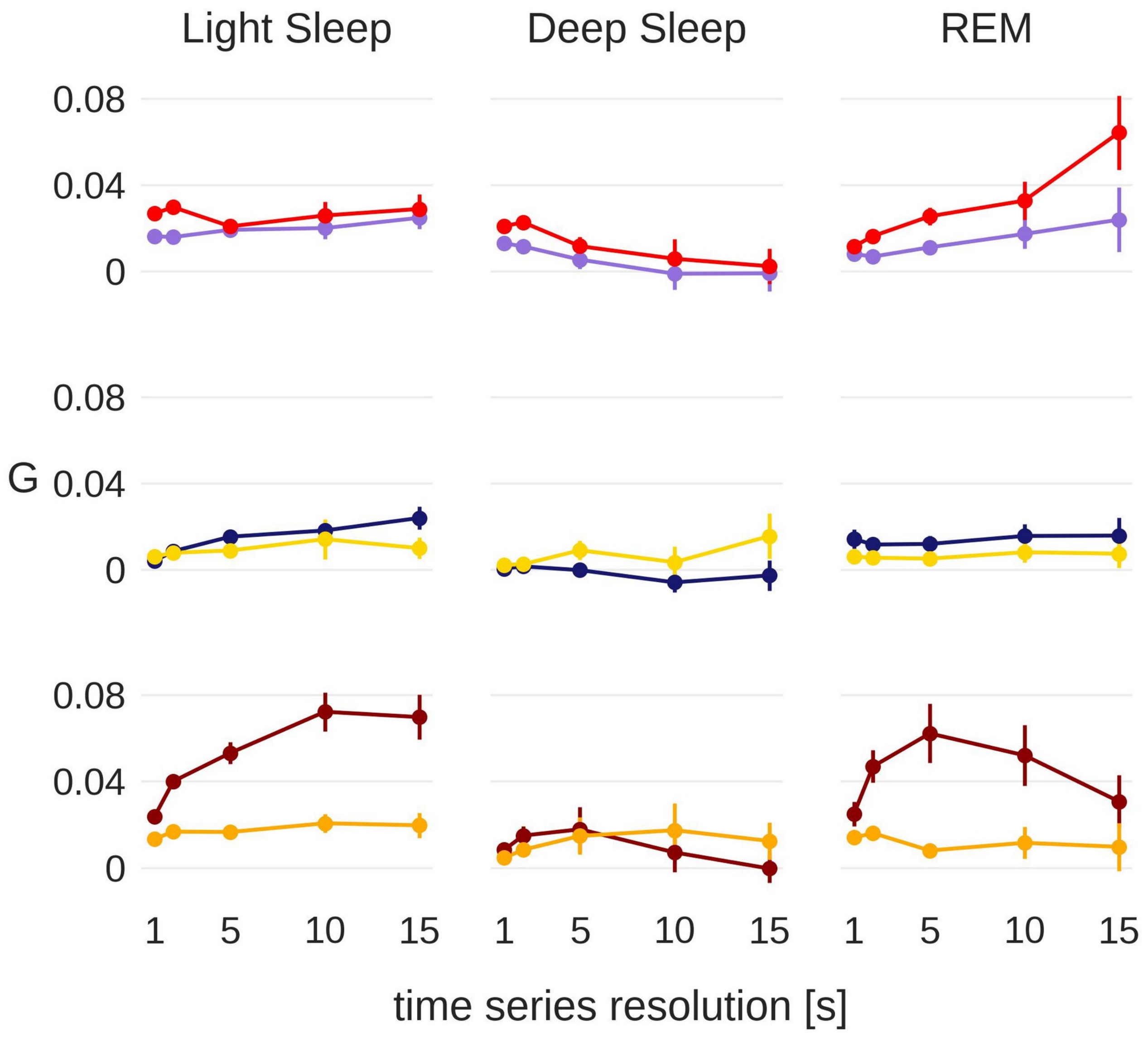

Fig. S6(b): Same as Fig. 6(b) (EC group), but only including male subjects.

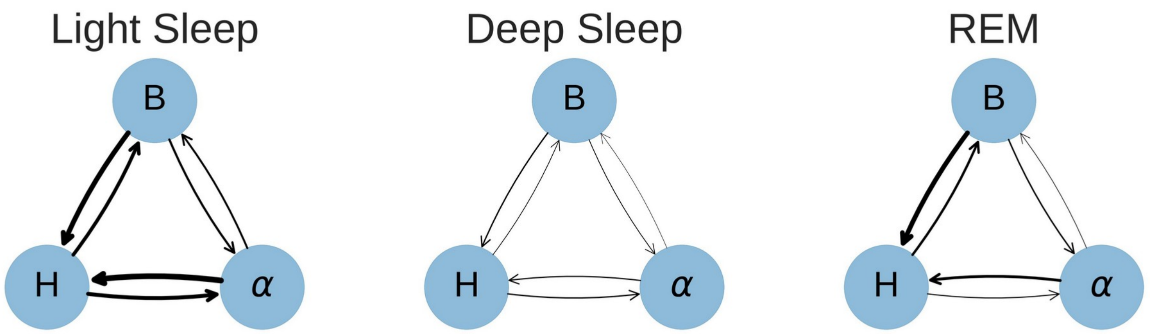

Fig. S7(a): Same as Fig. 6(a) (YC group), but only including male subjects.

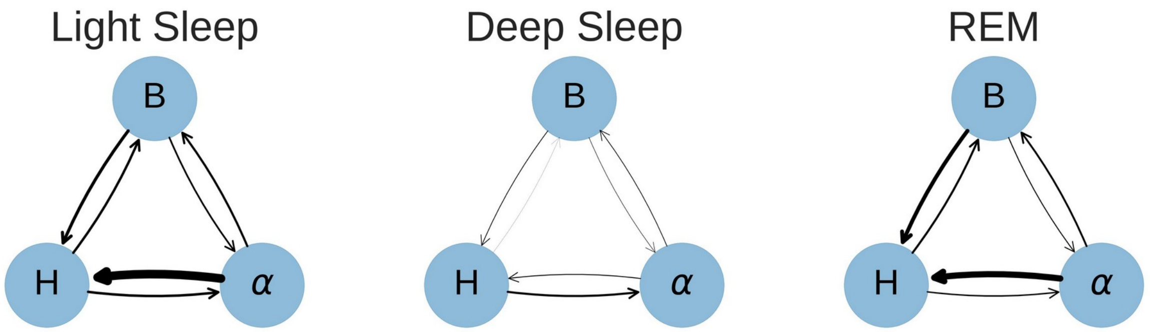

Fig. S7(b): Same as Fig. 6(b) (EC group), but only including male subjects.
